# Lilium leichtlinii subsp. maximowiczii (Regel) J.Compton (Liliaceae): a new combination for Maximowicz’s orange lily

**DOI:** 10.3897/phytokeys.174.62059

**Published:** 2021-03-12

**Authors:** James A. Compton

**Affiliations:** 1 Spilsbury Farm, Tisbury, SP3 6RU, UK Unaffiliated Tisbury United Kingdom

**Keywords:** *Lilium leichtlinii*, nomenclature, taxonomic conspectus, typification

## Abstract

The newly-proposed Lilium leichtlinii subsp. maximowiczii (Maxim.) J.Compton recognises the wide distribution of Maximowicz’s lily and provides long-term stability of the name. Lectotypes are designated for the names *Lilium leichtlinii* Hook.f., *L. maximowiczii* Regel, L. maximowiczii var. tigrinum Regel, *L. pseudotigrinum* Carrière and L. tigrinum var. lishmannii T.Moore.

## Introduction

*Lilium leichtlinii* Hook. f. was first described by Joseph Dalton Hooker of Kew. His description of this elegant lily was based on a citron-yellow flowered plant with strongly recurving perianth segments spotted with dark brownish-purple as shown clearly in the accompanying painting by Walter Hood Fitch (Hooker 1867: t. 5673).

*Lilium leichtlinii* is endemic to a disparate range of localities in Honshu and the Ryukyu Islands, Japan (Hayashi 2016: 113). It is in all probablility a recessive expression of a widespread orange flowered species that occurs in China, Korea, Japan and along the southern seaboard of the Russian Far East. The orange flowered lily has been given a number of names at the ranks of species, varietas and forma which are discussed below.

Morphologically, there is little to segregate the yellow and orange flowered lilies that belong in *L. leichtlinii* other than the colour of their floral organs. Both have floccose hairs on their pedicels, buds and on the perianth segments at the apices and median lines externally.

The purpose of this paper is to establish the name L. leichtlinii subsp. maximowiczii (Regel) J.Compton. Currently, it is widely known and accepted worldwide under the varietal name L. leichtlinii var. maximowiczii (Regel) Baker (Baker 1871) and is recognized under that name in both the horticultural literature (e.g. Haw 1986: 116); floristic publications (e.g. Hayashi 2016: 113) and in molecular phylogenies (e.g. Kim, Lim and Kim 2019: 2.1). At the rank of variety, however, it is predated by L. maximowiczii var. tigrinum Regel (Regel 1870). Under the rules of the ICN (Turland et al. 2018), names only have priority within their own rank and L. maximowiczii var. tigrinum still has priority even though it is attached to a superfluous, but valid, species name (see ICN Art. 11.2 ex. 4). Furthermore, additional use of the epithet L. leichtlinii var. tigrinum (Regel) G.Nicholson (1885), as cited in, for example, Ohwi (Ohwi 1984: 297), would inevitably engender much confusion with the morphologically-similar species *L. lancifolium* Thunb. (Thunberg 1794: 333) which was consistently and widely-known under the synonym *L. tigrinum* Ker Gawl. (Ker 1809) for more than two centuries and is still universally referred to today as the “Tiger lily”.

*Lilium lancifolium* is readily distinguished from *L. leichtlinii* by the production of dark purple bulbils formed in the leaf axils along the inflorescence axis. These are not found on the inflorescence axis in *L. leichtlinii*. Moreover, *L. leichtlinii* has bulbs which frequently send out underground stolons which produce axillary bulbils, a habit that does not occur in *L. lancifolium*. There is, however, an additional element of possible confusion between *L. lancifolium* and *L. leichtlinii*, the rare occurrence in Kyushu of the yellow flowered tiger lily L. lancifolium var. flaviflorum Makino (Makino 1933). This variety can also be distinguished from *L. leichtlinii* by the presence of purple stem bulbils produced in the leaf axils.

In recent phylogenetic studies, based on plastid and nuclear DNA sequence data, *L. lancifolium* has been shown to belong on a clade with *L. maculatum* and *L. pensylvanicum*, whereas *L. leichtlinii* has been shown to belong with *L. callosum* and *L. concolor* (Dubouzet and Shinoda 1999; Givnish et al. 2020).

## Typification of Leichtlin’s yellow lily

This lily was sent from Japan during the decade of Japanese history known as ‘bakumatsu’ or ‘end of the curtain’. Japan had finally ended its three centuries of isolationist ‘sakoku’ or ‘locked in’ period under the Tokugawa shoguns and opened up to trade with foreign nations under the government of the re-instated Meiji emperor. Little in the way of lily introductions from Japan to Europe or North America had occurred since von Siebold’s employment by the Dutch in Japan from 1823 to 1829. Siebold’s activities were limited almost exclusively to the surrounding countryside around Nagasaki on Kyushu Island and specifically to the little artificial Island of Dejima (Compton and Thijsse 2013).

Siebold had been responsible for sending back many good garden-worthy plants from Japan and was particularly fond of the Japanese lilies. He included a number of them in his Prix Courants and Catalogues Raisonnés from plants he cultivated in his Leiden garden. He encountered this lily on his second visit to Japan from 1859 to 1862 when he returned to Japan as a trade envoy for the Dutch Government during the bakumatsu. Although Siebold did not give this yellow flowered lily a Latin name, he had it painted in 1861 on fine Japanese paper by Shimizu Higashiya under the Japanese name Kihirato yuri (Siebold’s Florilegium vol. 1b, Pl. 299; vol. 2 no. 830). The painting, which is kept in the Russian Academy of Sciences Library in St. Petersburg, was annotated in pencil ‘*Lilium* spec. nov.’ by Siebold and on the verso bottom left in pencil by Maximowicz ‘*Lilium testaceum*’. The latter annotation is probably Maximowicz’s identification of it as the lily described by Lindley (1842: 51; Lindley 1843 t. 11) as the “Yellow Japan Lily” under the name *L. testaceum* Lindl. This curious lily was in cultivation at the nursery of William Rollison & Sons of Upper Tooting near London and is now known to be a hybrid of two European species (Sterling 2017: 202).

Sir Joseph Dalton Hooker described *Lilium leichtlinii* after it had been introduced to England in a batch of bulbs of *L. auratum* Lindl., which itself had only been described as a new species five years earlier. The bulbs had been sent from Japan to the nursery of James Veitch & Sons of Chelsea. Hooker does not say who had sent the bulbs, but the shipment could have been arranged through a local supplier by John Gould Veitch who was in Japan from 1861–1862 from where he is recorded to have sent back bulbs of *L. auratum* (Veitch 1906: 50).

Hooker named the yellow flowered species after Maximilian Leichtlin (1831–1910), a keen horticulturist and bulb enthusiast who corresponded with other like-minded people including lily monographer Henry John Elwes (see below). Another of Leichtlin’s correspondents was John Gilbert Baker who had joined the herbarium and library staff at Kew in 1866. Max Leichtlin, born in Karlsruhe in southwest Germany, had worked for two years in the Van Houtte nursery near Ghent before founding his own private botanic garden in Baden-Baden, Germany. There he grew many bulbous plants, including Hooker’s recently described *L. leichtlinii* and another he recorded in his list of cultivated plants as L. leichtlinii var. major (Leichtlin 1873: 10). The latter plant, although undescribed by him, might refer to the taxon described in the same year as *L. leichtlinii* [unranked] *majus* G.F.Wilson (Wilson 1873: 371) and later as L. leichtlinii f. majus G.Nicholson, who stated that this yellow-flowered, purple-black spotted form was luxuriant and attained a height of 5 ft [1.52 m] and that it had been introduced from Japan in 1872 (Nicholson 1885: 270).

There is a sheet at K which is partitioned into three different sections. On the right hand section with the barcode K-000464728, the upper right hand portion consists of a pedicel and three leaves along with a dissected flower in a herbarium capsule and bears a label with “Lilium leichtlinii Hk fil. Hort. Barr July 30 1872”. Below that is a cut out illustration in pencil of two bulbs, the drawing bearing the legend “2 & 3 Lilium leichtlinii nat. size. From bulbs cult. by Colchester Bulb Company. Comm. F. Burbidge February 1877”. The Barr specimen and the Burbidge drawing are both added on to this sheet after Hooker’s protologue and are, therefore, not original material. The history of these two additions on the sheet may be of minor interest. Peter Barr was a daffodil specialist who, with his business partner Edward Sugden, owned a shop in King Street, St. James’s, London, England from 1861. *Lilium leichtlinii* was listed as a new entrant in their 1871 catalogue for the very expensive price of “ten shillings and sixpence” [equivalent to ca. 600 GBP or 830 USD today]. Frederick William Burbidge was employed at Kew principally as a draughtsman from 1868 to 1870. He then became a plant collector in Borneo for James Veitch’s nursery. Although Burbidge communicated the bulb sketches to Kew (probably to Joseph Hooker) in 1877, it is possible that he drew them earlier.

The whole left hand portion of the sheet with a stamp for Herbarium Hookerianum 1867 on it and with the modern barcode K-000464729 is taken up with two stems, one with a single flower and has a label with “Lilium leichtlinii? Fl. July 5673. Bot. Mag. From Mr Fitch, Accpt. Veitch 1867. Japan”. There is also an accompanying letter from the Royal Exotic Nursery, King’s Road, Chelsea, London dated 24 July 1867 from Veitch which includes the following information: “Dear Mr Hooker, Amongst the imported *Lilium auratum* roots, which came home last winter we have found one now in bloom which seems to us quite different from any other kind we have seen, in fact, more like a yellow Turk’s Cap lily. We send you the flower by bearer and should be glad to know if you consider it new and worth figuring”. The annotation on the label with the number “5673”, “Bot. Mag.”, “Fl. July” and “from Mr Fitch” are direct references to the illustration by Walter Hood Fitch that accompanied Hooker’s protologue dated 1 November 1867 in *Curtis’s Botanical Magazine* (Hooker 1867: t. 5673). In his protologue, Hooker mentions that the lily was “communicated to me in July of the present year”. There are, therefore, two elements that accompany the protologue, the sheet at K and the illustration in the Bot. Mag. The portion of the sheet barcoded K-000464729 is undoubtedly original material and is eligible to be the lectotype, if not the holotype for the name (Art. 9.3, Turland et al. 2018). http://specimens.kew.org/herbarium/K000464729

## Naming and typification of the orange lily

The first publication of *Lillium leichtlinii* with orange rather than yellow flowers was, in fact, on exactly the same day i.e. 1 November 1867 as the publication of the yellow-flowered species named *L. leichtlinii* by Joseph Hooker (J. McNeill pers. com.). Precedence for the use of *L. leichtlinii* (Art. 11.5 of the ICN), however, was provided by Baker who chose *L. pseudotigrinum* as a synonym of L. leichtlinii var. maximowiczii (Regel) Baker (Baker 1871: 1422).

*Lilium pseudotigrinum* Carrière was named by Elie-Abel Carrière, a Parisian horticulturist as the false tiger lily (Carrière 1867: 411). He named it specifically in contrast to *Lilium lancifolium* Thunb. which was known at that time under the later synonym *L. tigrinum* Ker Gawl. Morphologically *L. pseudotigrinum* has all the characters that equate it to *L. leichtlinii*, but with orange rather than yellow flowers and it has, consequently, been combined within that species at various ranks.

Carrière included a painting by F. Yerna in his protologue of *L. pseudotigrinum* (Carrière 1867: t. “*Lilium pseudotigrinum*”). Yerna’s illustration was painted from a plant cultivated in the Muséum [national d’histoire naturelle] de Paris, also known as the Jardin des Plantes. Carrière stated that the plant had been introduced from China, but he did not mention who had collected it or its precise locality. There were a number of French botanists who collected seeds and bulbs in eastern China and sent them back to Carrière in Paris in the early 1860s, following the Treaty of Tientsin [Tianjin] in 1858. One such was Gabriel Eugène Simon (1829–1896), a French diplomat who travelled extensively in Hebei and Jilin Provinces where the lily occurs in the wild and who is known to have sent seedlings of *Prunus simonii* (Decne) Carrière back to the Paris Muséum in the 1860s. Yerna’s illustration clearly shows the flowers of the orange flowered *L. leichtlinii* with their dark brown speckling and anthers with brown pollen. As there are no garden records for the living collections at P for that time and, as there is no evidence of any herbarium specimen in P that could refer to Carrière’s plant (Florent Martos pers. comm.), Yerna’s illustration is chosen here as the lectotype for the name.

A year later, Eduard August von Regel also described the orange flowered *L. leichtlinii* as *L. maximowiczii* from one of Maximowicz’s collections in Japan (Regel 1868a: 26). Regel described the lily in the supplement to the 1866 Index Seminum of the Imperial Botanic Garden in St. Petersburg, published in 1868, in which he mentioned the distinctive scarlet-orange sulcus or groove in the perianth segments and their being dotted with dark purple from their centres towards the base. Later that same year, Regel included another description of *L. maximowiczii* with an illustration in *Gartenflora*, the journal he edited and had founded in 1852 (Regel 1868b: 322, t. 596). The illustration was painted from a plant introduced from a garden in Japan collected by Carl Johann Maximowicz between 1860 and 1864 and then cultivated in the Imperial Botanical Garden, St. Petersburg.

**Figure 1.**
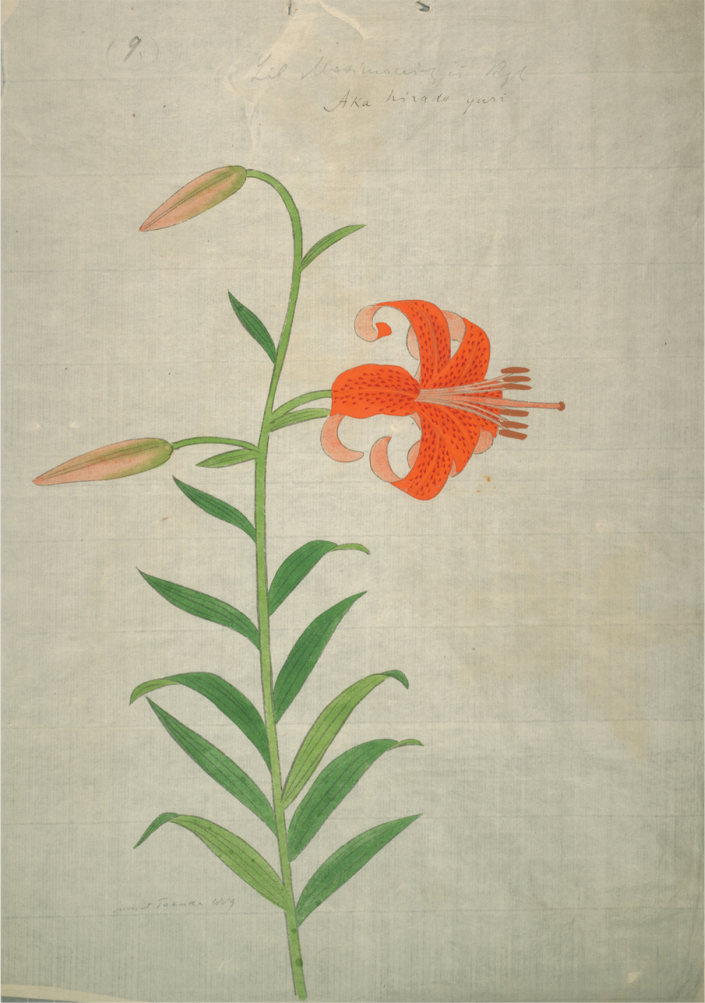
Watercolour by an unknown Japanese artist entitled “Aka hirado yuri” depicting Lilium leichtlinii subsp. maximowiczii (Regel) J.Compton.

Amongst the collections in the Herbarium at St. Petersburg (LE) is a fine watercolour on paper of this orange flowered lily annotated in pencil above the lily “9 Lil maximowiczii Rgl” and in ink “Aka hirado yuri” by an unknown hand (Fig. 1). Below on the left is also written in pencil “misit Tokuda 1889”. The sender is very likely to have been the botanist Shôzô Tokuda who had participated in the International Congress of Botany and Gardening in St Petersburg in 1884 and while there, had helped Maximowicz clarify the Japanese localities of several of his collections (Grabovskaya-Borodina 2016: 63). The artist of the illustration is unknown and the work is undated; however, as Tokuda sent the illustration to Maximowicz in 1889, it cannot be material that could have been the basis for either of the illustrations in *Gartenflora* referring to either *L. maximowiczii* or L. maximowiczii var. tigrinum (Regel 1868b; 1870).

There are also eight sheets in LE labelled *Lilium leichtlinii* that were collected in Japan near Yokohama in 1862 by C. J. Maximowicz, five of them bearing the annotations ‘cult.’ or ‘cultivatus’. These all have the clear paleness on drying of the yellow flowered species described by Hooker (1867) and are all probably of cultivated origin. In addition, there are six collections by Maximowicz labelled *Lilium maximowiczii* and a further eight collections similarly labelled that were collected by Maximowicz’s Japanese assistant Sugawa Tschonoski [Chonosuke] all collected between 1862 and 1864 in Japan. Regel specifically mentioned that *L. maximowiczii* was based on material cultivated in the Imperial Botanic Garden in St. Petersburg and therefore, none of these specimens can be considered as type material of *L. maximowiczii*.

**Figure 2.**
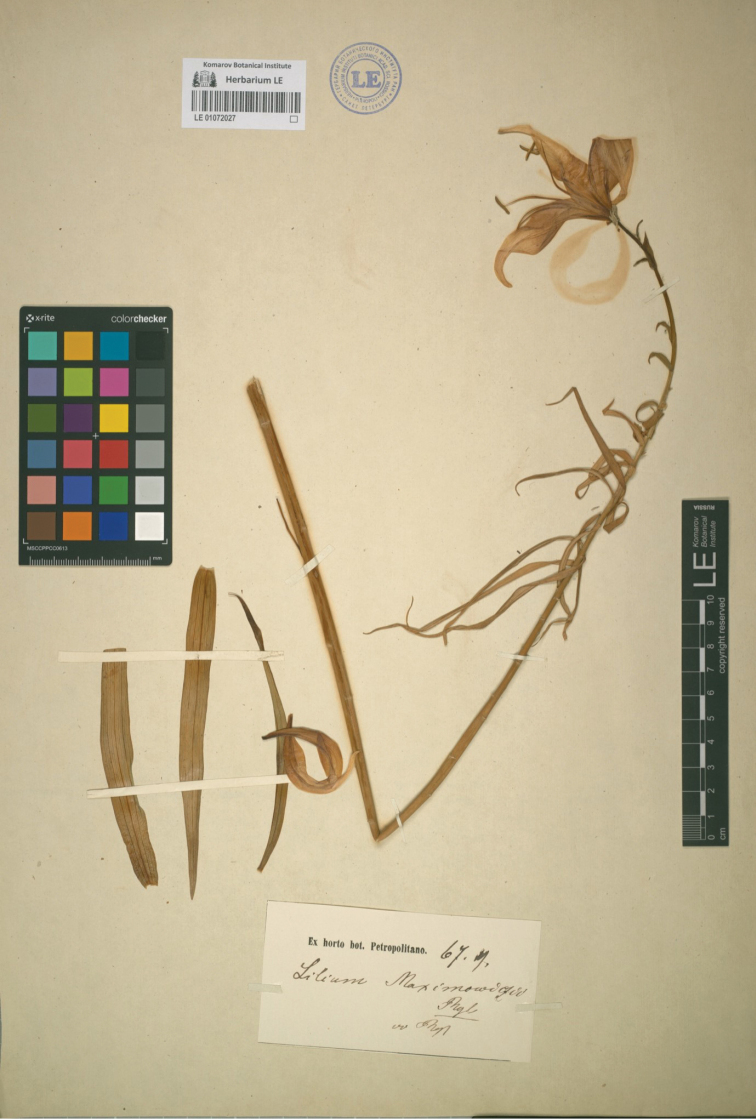
Lectotype sheet LE-01072027 of Lilium leichtlinii subsp. maximowiczii (Regel) J.Compton.

There are however, three sheets annotated by Regel “L. maximowiczii” with the printed label ‘Ex horto bot. Petropolitano’. One is dated 68.6 with “teste Rgl” [according to Regel] indicating that it was gathered in June 1868. The sheet consists of a few scattered tepals, some floral dissections and some leaves (LE-01072026). Another is dated 67.7 indicating July 1867 with the addition of “vv Rgl” [vidi vivit], stating that Regel had seen the living plant (LE-01072027), the stem having a single open flower and three basal leaves. The third with a flowerless stem and a herbarium capsule with some seeds in it, has the additional annotations “fl. Punctato, 6. x. 71 v.v Maxim.” (LE-01072028). This last specimen is dated 6 October 1871. Although it was seen by Maximowicz, it was collected after the dates of the two publications and is, therefore, not original material. The other two, however, can be considered as original material. Bearing in mind that Regel specifically mentioned material that was cultivated in the St Petersburg Garden in his protologue and, in consideration of the time needed then for the process of publication, it would be wise to choose the earlier of these specimens gathered from the Garden in 1867 (LE-01072027), as the lectotype for the name (Fig. 2). The other specimen can be considered to be a paratype.

Two years later, another of Maximowicz’s collections from Japan flowered in the Imperial Garden and was figured again by Regel in *Gartenflora*. This he called L. maximowiczii var. tigrinum Regel. It was distinguished from his earlier species description by being more upright, having narrower leaves and flowers with perianth segments that recurved only at the tip and with blackish-purple speckling (Regel 1870: 290). In all respects, these can be considered to be merely minor variations of what he had already described. As far as selecting material for typification purposes, there is no direct link to any of the herbarium specimens in LE that Regel saw and/or annotated that links them with the varietal name. The illustration in *Gartenflora* (Regel 1870: t. 664), therefore, must be considered as the only original material available as the lectotype for that name.

Lilium tigrinum var. lishmannii T.Moore was described briefly under the name *L. lishmanni* by R. D. [Richard Dean] in *Florist and Pomologist* 1872: 259 where it was awarded a First Class Certificate at the Royal Horticultural Society’s meeting in South Kensington, London on 24 August of that year. It was said to “represent a fine variety of the tigrinum type, with large dull-red flowers profusely spotted with black in a very distinct manner” and that it had originated from Mr T. R. Tuffnell of Uxbridge (R.D. 1872: 259). As Dean described it as a variety that was not attached to any species, it is not considered to be validly published (Art. 11.4 and Art. 24.1) and is, in any case, merely a dark-flowered horticultural variant of L. leichtlinii subsp. maximowiczii.

The following year, the editor of *Florist and Pomologist* and curator of the Society of Apothecaries Garden in Chelsea [Chelsea Physic Garden], Thomas Moore, formally recognised this lily with an accompanying illustration and a full description as a variety of the tiger lily (Moore 1873: 16). The illustration plate 2 shows a stem with tawny orange-red flowers with dark blackish spotting. Clearly shown are also the papillose margins of the orange nectaries. This illustration was painted from Tuffnell’s plant, cited by Dean the year before who had received the plant in 1871 from Mr. Lishmann in Japan. Moore clearly believed that this lily actually belonged to the Tiger lily which was then almost universally known as *L. tigrinum*, [now *L. lancifolium*] which also occurs in Japan. The stem in the illustration, however, is green and scabrid, not dark purple and there are no stem bulbils present. It is clearly a dark orange-red flowered variant of *L. leichtlinii*.

Between March 1877 and May 1880, Henry John Elwes produced his magnificent monograph of the genus *Lilium* (Elwes 1877–1880). In that work, he included the yellow-flowered *Lilium leichtlinii* as plate 39 in part three of his published monograph for August 1877, with a plate by Walter Hood Fitch. In the text, he attempted to distinguish between *L. leichtlinii* and what he later portrayed as *L. maximowiczii*. Elwes included a short table given to him by Maximowicz consisting of two columns showing the distinguishing diagnostic characters of the two lilies. Elwes also added that these characters were, in his opinion, not very stable and he believed that *L. maximowiczii* should be regarded as a variety of *L. leichtlinii*, following Baker’s earlier recombination (Baker 1871).

Elwes later included an illustration of *L. maximowiczii* as plate 40 in part six of his monograph for January 1879 (Elwes 1877–1880). The plate painted by Walter Hood Fitch, included four examples of the variation he had seen within the orange lily. These comprised *L. maximowiczii* with orange flowers and paler reverse; L. maximowiczii var. bakeri Elwes, with dark reddish-orange perianth segments speckled with dark purple and pale orange on the reverse; L. maximowiczii var. regeli Elwes, with dark reddish perianth segments and red streaking, pale orange on the reverse and *L. pseudotigrinum* orange with red speckles and a yellow reverse. These all clearly belong within the circumscription of the subspecies proposed here. It is clear too that Elwes’s recognition of var. bakeri and var. regelii are merely floristic colour variants and are here best regarded as horticultural cultivars ‘Bakeri’ and ‘Elwesii’.

## Taxonomic Conspectus

### Lilium leichtlinii

Taxon classificationPlantaeLilialesLiliaceae

Hook.f., Bot. Mag. 93 t. 5673 (1867).

CACC428F-5900-5A44-B9DE-BD713288A13F

#### Lectotype.

Designated here: Japan, herbarium Hookerianum 1867 “from Mr Veitch, Japan, received 1867” fl? July. 5673. Bot. Mag.” [K-000464729] (K, lecto.!)

##### Key to subspecies of *Lilium leichtlinii*

**Table:** 

1	Flowers with perianth segments yellow, filaments and style pale yellow, Japan	**subsp. leichtlinii**
2	Flowers with perianth segments orange to dark brownish-red, filaments and style pale pinkish-orange, China, Japan, Korea, Russia	**subsp. maximowiczii**

### Lilium leichtlinii subsp. leichtlinii

Taxon classificationPlantaeLilialesLiliaceae

145B71F5-0E88-5E22-8480-036F5D088962

= Lilium leichtlinii [unranked] majus G.F.Wilson, J. Hort. Cottage Gard. n.s. 25: 371 (1873)
≡ Lilium leichtlinii var. majus (G.F.Wilson) Baker, J. Linn. Soc., Bot. 1874: 248 (1874)
≡ Lilium leichtlinii f. majus (G.F.Wilson) G.Nicholson, Ill. Dict. Gard. 2: 270 (1885).

#### Note.

Although Nicholson refers to the various varieties of lilies in his introduction to his entry on *Lilium*, he distinctly states in his description of *L. leichtlinii majus* “this is a luxuriant form” (Nicholson 1885: 270).

#### Description.

***Bulb*** subglobose to globose 2–4 × 2–4 cm forming short subterranean stolons with bulbils, scales white, ovate, thick; ***stem*** 40–180 cm, green, slightly scabrid or floccose- tomentose, especially on upper inflorescence axis; ***leaves*** scattered, sessile, linear, 8–16 × 0.5–1.2 cm, glabrous or slightly white floccose, especially beneath, margins tomentose when young; ***inflorescence*** 1–5 flowered, ***pedicels*** 10–18 cm long, glabrous to slightly floccose; ***flowers*** pendulous, citron-yellow, lightly to heavily dotted with purplish-brown speckles from the middle portion of the tepal to the base, rarely covered in purplish-brown streaks, not fragrant, tepals pubescent at apex and below, strongly recurved 5–8 × 1–2 cm lanceolate, apex subacute, nectaries yellow, margins raised, papillose; ***stamens*** glabrous, filaments yellow, spreading outwards, anthers brown, pollen yellow; ***Style*** pale yellow, upwards curving, stigma reddish-brown, lobes short; ***capsule*** ellipsoid 3–5 cm long.

#### Distribution.

Japan, Honshu (Akika Pref., Shizuoka Pref.); Ryukyu Islands (Amami-o-shima).

#### Ecology.

Growing in open rich marshy meadows, along stream margins and in sandy terrain amongst low scrub, sea level to 1300 m of elevation. Flowering in July and August. Germination epigeal.

#### Illustration.

https://rhslilygroup.org/2019/wp-content/uploads/2018/11/L-leichtlinii-lechtlinii.jpeg

### Lilium leichtlinii subsp. maximowiczii

Taxon classificationPlantaeLilialesLiliaceae

(Regel) J.Compton, comb. et stat. nov.

87E92E79-60D6-56DF-9164-42599B8DBF91

urn:lsid:ipni.org:names:77215715-1

Basionym: Lilium maximowiczii Regel, Ind. Sem. Hort. Petrop. 1866 Suppl. Annot. Bot. VI: 26 (1868a). Lectotype designated here (Fig. 2): ex Japan cult. Hort. Petrop. July 1867. *C.J.Maximowicz s.n*. 1862–1864 [LE-01072027] (LE, lecto.!); Syntype: ex Japan cult. Hort. Petrop. July 1867. *C.J.Maximowicz s.n*. 1862–1864 [LE-01072026] (LE, syn.!)
≡ Lilium leichtlinii var. maximowiczii (Regel) Baker, Gard. Chron. 1871(2): 1422 (1871)
= Lilium pseudotigrinum Carrière, Rev. Hort. 1867: 411 (1867). Lectotype designated here: Cult. (P) ex China without collector [Icon] Rev. Hort. 1867: t. L. pseudotigrinum.
≡ Lilium leichtlinii var. pseudotigrinum (Carrière) Baker, J. Roy. Hort. Soc. n.s. 4(13): 47 (1873)
≡ Lilium maximowiczii var. pseudotigrinum (Carrière) Elwes, Monogr. Lilium (1879)
≡ Lilium leichtlinii f. pseudotigrinum (Carrière) Hara & Kitam., Acta Phytotax. Geobot. 36(1–3): 93. (1985)
= Lilium maximowiczii var. tigrinum Regel, Gartenflora 19: 290, t. 664, fig. 4 (1870) Lectotype designated here: [Icon] Gartenflora 19 t. 664, fig. 4 (1870).
≡ Lilium leichtlinii var. tigrinum (Regel) G.Nicholson, Ill. Dict. Gard. 2: 271 (1885)
– Lilium maximowiczii f. tigrinum Leichtlin, Pflanzen-Sammlung des Leichtlin’schen Gartens in Baden-Baden (1873: 11) nom. nud.
= Lilium tigrinum var. lishmannii T.Moore, Florist & Pomol. 1873: 16, tab.2 (1873). Lectotype designated here: [Icon] Florist & Pomol. 1873: tab.2 (1873).
– L. tigrinum var. jocundum Tilton, Bailey Standard Cycl. Hort. 2: 1870 (1933) nom. nud.

#### Diagnosis.

Differing from subsp. leichtlinii in the following characters: ***bulb*** with stolons that can extend to 2 m; ***stem*** green, purple streaked or spotted (vs. stem green unspotted); ***leaves*** linear or lanceolate to 1.6 cm wide (vs. 0.5–1.2 cm wide); ***Inflorescence*** with (2) –5–12 flowers, ***flowers*** light orange or reddish-orange with dark reddish-brown speckling, rarely reddish-brown streaking (vs. yellow), nectaries with orange-red papillae marginally (vs. yellow); stamens with pale orange filaments (vs. pale yellow), anthers brown, pollen reddish-orange (vs. yellowish-brown); ***style*** pale orange (vs. pale yellow).

#### Distribution.

China (Hebei, Jilin, Liaoning, Shaanxi); Japan, Honshu, Kyushu, northern Ryukyu Islands, Shikoku; North Korea (North Hamgyeong); Russian Far East (Primorskiy Kray); South Korea (Chungchungbook, Gangwon, Gyunggi).

#### Ecology.

Growing in open rich marshy meadows, along stream margins and in sandy terrain amongst low scrub, sea level to 1300 m. of elevation. Flowering in July and August. Germination epigeal. It is worth noting that both diploid and triploid plants have been found in Korea and that plants have been observed to produce subterranean stolons that extended to as much as 2 m in length (Kim et al. 2016: 104).

#### Illustration.

https://rhslilygroup.org/2019/wp-content/uploads/2018/11/L-leichtlinii-maximoviczii.jpeg

## Conclusion

The yellow flowered *Lilium leichtlinii* has been known for centuries in Japan as the ki-hirato yuri or ‘lily of the sun’ and the orange flowered as the ‘ko-oni yuri’, the latter being known under a range of local names in Chinese, Korean and Russian. Naming of the orange lily is compounded, in this case, by the fact that the yellow “species” is almost certainly a recessive variant restricted to a few isolated populations in Japan. The orange-red flowered subspecies represents a much more widespread species. The nomenclatural problem of the varietal epithet L. leichtlinii var. maximowiczii being superseded by L. maximowiczii var. tigrinum (see ICN Art. 11.2 ex. 4, Turland et al. 2018) is resolved by the recognition of the orange flowered taxon as L. leichtlinii subsp. maximowiczii.

## Supplementary Material

XML Treatment for Lilium leichtlinii

XML Treatment for Lilium leichtlinii subsp. leichtlinii

XML Treatment for Lilium leichtlinii subsp. maximowiczii
